# Supplementary material for: Effects of a High-Fat Diet on Insulin-Related miRNAs in Plasma and Brain Tissue in APPSwe/PS1dE9 and Wild-Type C57BL/6J Mice
Source: Nutrients. 2024 Mar 26;16(7):955. doi: 10.3390/nu16070955 (PMC11013640; doi:10.3390/nu16070955)
Supplement: Supplementary file 1 [file nutrients-16-00955-s001.zip › Supplementary_Material_HFD.pdf]

Supplementary Table S2 | Significant enriched KEGG pathways for the miRNAs linked to neurodegeneration.

KEGG ID	KEGG term	Gene Set Size	Counts	Padj	Genes
Mmu05206	MicroRNAs in cancer	142	58	<0.001	*Apc, Bak1, Bcl2, Bcl2l11, Bcl2l2, Bmf, Bmpr2, Ccnd1, Ccnd2, Ccne1, Ccne2, Ccng1, Cdc25a, Cdc25c, Cdk6, Cdkn1a, Crk, Crkl, Ddit4, Dicer1, Dnmt3a, E2f3, Ep300, Erbb3, Gls, Hmox1, Kif23, Mcl1, Mdm4, Mmp16, Notch1, Notch2, Nras, Pdcd4, Pdgfrb, Prkca, Prkcb, Pten, Raf1, Rps6ka5, Rptor, Shc1, Sirt1, Slc7a1, Socs1, Sos1, Stat3, Stmn1, Tnc, Tpm1, Trim71, Trp63, Ube2i, Vegfa, Wnt3, Wnt3a, Zeb2, Zfpm2*
Mmu04919	Thyroid hormone signaling pathway	115	44	<0.001	*Actb, Akt3, Atp1a2, Atp1b1, Atp2a2, Ccnd1, Ctnnb1, Dio2, Ep300, Esr1, Foxo1, Hdac2, Hif1a, Itgav, Kat2b, Mapk1, Med1, Med13, Med13l, Med17, Ncoa2, Ncoa3, Ncor1, Notch1, Notch2, Nras, Pfkp, Pik3ca, Pik3cb, Pik3cd, Pik3r3, Plcb1, Pln, Prkca, Prkcb, Raf1, Rcan2, Rheb, Rxra, Sin3a, Slc9a1, Stat1, Tbc1d4, Wnt4*
Mmu04070	Phosphatidylinositol signaling system	98	35	<0.001	*Calm1, Cds1, Cds2, Dgkb, Dgke, Dgkk, Impa1, Inpp5e, Ip6k3, Ipmk, Ippk, Itpk1, Itpkb, Itpkc, Mtmr2, Mtmr4, Mtmr6, Ocrl, Pi4k2a, Pi4k2b, Pik3c2a, Pik3ca, Pik3cb, Pik3cd, Pik3r3, Pikfyve, Pip4k2b, Plcb1, Ppip5k1, Ppip5k2, Prkca, Prkcb, Pten, Sacm1l, Synj1*
Mmu04010	MAPK signaling pathway	294	101	<0.001	*Akt3, Araf, Arrb1, Atf2, Bdnf, Cacna1c, Cacna1e, Cacna2d1, Cacna2d2, Cacnb1, Cacnb2, Cacnb4, Cacng7, Crk, Crkl, Csf1, Csf1r, Dusp16, Dusp6, Dusp7, Efna1, Erbb3, Erbb4, Ereg, Fgf10, Fgf18, Fgf2, Fgf9, Fgfr1, Fgfr2, Flna, Flnb, Flnc, Flt1, Flt3, Hgf, Igf1r, Igf2, Il1rap, Irak1, Jun, Jund, Kit, Kitl, Lamtor3, Map2k3, Map2k7, Map3k1, Map3k12, Map3k13, Map3k14, Map3k2, Map3k3, Map3k5, Mapk1, Mapk14, Mapk8, Mapkapk2, Mapt, Max, Mecom, Mef2c, Mknk2, Nf1, Nfatc3, Nlk, Nras, Ntrk2, Pdgfrb, Ppp3ca, Ppp3cb, Ppp3r1, Prkca, Prkcb, Raf1, Rap1a, Rap1b, Rapgef2, Rasa1, Rasa2, Rasgrf2, Rasgrp1, Rela, Relb, Rps6ka2, Rps6ka3, Rps6ka4, Rps6ka5, Sos1, Srf, Stk3, Stmn1, Tab2, Taok1, Tgfb1, Tgfbr1, Tgfbr2, Tnf, Traf6, Vegfa, Vegfb*
Mmu04360	Axon guidance	175	56	<0.001	*Arhgef12, Bmpr2, Camk2a, Camk2d, Cxcl12, Cxcr4, Dpysl5, Efna1, Efnb2, Enah, Epha4, Epha7, Ephb3, Ephb4, Gnai1, Gnai2, Mapk1, Nck2, Nfatc3, Nras, Nrp1, Ntng1, Pak3, Pak6, Pak7, Pard6g, Pik3ca, Pik3cb, Pik3cd, Pik3r3, Plxna1, Plxna2, Plxna4, Plxnb3, Plxnc1, Ppp3ca, Ppp3cb, Ppp3r1, Prkca, Ptch1, Raf1, Rasa1, Robo2, Sema3a, Sema4b, Sema4c, Sema4f, Sema6a, Sema6b, Sema6d, Smo, Srgap3, Unc5b, Unc5c, Wnt4, Wnt5a*
Mmu04024	cAMP signaling pathway	198	56	<0.001	*Acox1, Adcy1, Adcy5, Adcy6, Adcy7, Adcy9, Adcyap1r1, Adrb1, Adrb2, Akt3, Atp1a2, Atp1b1, Atp2a2, Atp2b3, Bdnf, Cacna1c, Calm1, Camk2a, Camk2d, Camk4, Chrm2, Cngb3, Creb1, Creb3l2, Creb5, Ep300, Gnai1, Gnai2, Gria2, Gria3, Gria4, Grin2b, Hcn2, Hhip, Htr1a,*

					Jun, Mapk1, Mapk8, Npy1r, Pde3b, Pik3ca, Pik3cb, Pik3cd, Pik3r3, Pln, Ppara, Ppp1ca, Ptch1, Raf1, Rap1a, Rap1b, Rapgef4, Rela, Slc9a1, Tiam1, Vav3
Mmu04144	Endocytosis	270	72	<0.001	*Acap2, Acap3, Agap1, Ap2m1, Arap2, Arf6, Arfgef1, Arfgef2, Arpc1a, Arpc1b, Arpc5, Arrb1, Asap2, Bin1, Capza1, Cav2, Cav3, Cblb, Chmp1a, Chmp2b, Cltc, Cxcr4, Cyth1, Cyth3, Dnm3, Ehd4, Epn2, Eps15, Fgfr2, Git2, Grk4, Grk5, Grk6, H2-D1, Igf1r, Igf2r, Ist1, Itch, Kif5a, Kif5b, Kif5c, Ldlrap1, Mvb12b, Nedd4, Nedd4l, Pard6g, Pdcd6ip, Psd, Psd3, Rab11b, Rab11fip2, Rab5b, Sh3gl2, Smap1, Smap2, Smurf2, Snx5, Stam, Stam2, Tfrc, Tgfbr1, Tgfbr2, Traf6, Usp8, Vps26a, Vps26b, Vps37a, Vps4b, Wasl, Wipf1, Wipf2, Wwp1*
Mmu05202	Transcriptional misregulation in cancer	183	47	<0.001	*Aff1, Atf1, Bak1, Bcl11b, Bcl6, Bmp2k, Ccnd2, Ccnt2, Cd40, Cdk9, Cdkn1a, Csf1r, Dot1l, Dusp6, Erg, Etv1, Flt1, Flt3, Foxo1, Gria3, H3f3b, Hdac2, Hoxa10, Hoxa9, Id2, Igf1r, Igfbp3, Kmt2a, Maf, Max, Mef2c, Mllt3, Mycn, Ncor1, Nr4a3, Plat, Prom1, Rela, Runx1, Runx2, Rxra, Sin3a, Six1, Six4, Sp1, Tgfbr2, Tspan7*
Mmu05200	Pathways in cancer	528	133	<0.001	*Adcy1, Adcy5, Adcy6, Adcy7, Adcy9, Akt3, Apc, Appl1, Ar, Araf, Arhgef12, Bak1, Bcl2, Bcl2l11, Bmp2, Calm1, Camk2a, Camk2d, Ccdc6, Ccnd1, Ccnd2, Ccne1, Ccne2, Cdk6, Cdkn1a, Col4a1, Col4a2, Col4a3, Col4a4, Crk, Crkl, Csf1r, Csf2rb, Ctnna1, Ctnnb1, Cxcl12, Cxcr4, Dll1, E2f3, Egln2, Egln3, Ep300, Epas1, Esr1, Ets1, Fgf10, Fgf18, Fgf2, Fgf9, Fgfr1, Fgfr2, Flt3, Foxo1, Frat2, Fzd6, Gli2, Gnai1, Gnai2, Gnaq, Hdac2, Hes1, Hgf, Hhip, Hif1a, Hmox1, Ifng, Ifngr1, Igf1r, Igf2, Il15ra, Il6ra, Il7r, Itga6, Itgav, Jag1, Jun, Kit, Kitl, Lpar3, Lrp6, Mapk1, Mapk8, Max, Mecom, Ncoa3, Nos2, Notch1, Notch2, Nras, Pdgfrb, Pik3ca, Pik3cb, Pik3cd, Pik3r3, Plcb1, Pmaip1, Prkca, Prkcb, Ptch1, Pten, Raf1, Ralb, Ralgds, Rarb, Rasgrp1, Rela, Rps6ka5, Rps6kb1, Runx1, Rxra, Skp1a, Smad4, Smo, Sos1, Sp1, Stat1, Stat2, Stat3, Sufu, Tcf7, Tgfb1, Tgfbr1, Tgfbr2, Traf3, Traf4, Traf6, Vegfa, Vegfb, Wnt1, Wnt3, Wnt3a, Wnt4, Wnt5a*
Mmu04151	PI3K-Akt signaling pathway	357	89	<0.001	*Akt3, Atf2, Bcl2, Bcl2l11, Bdnf, Ccnd1, Ccnd2, Ccne1, Ccne2, Cdk6, Cdkn1a, Chrm2, Col4a1, Col4a2, Col4a3, Col4a4, Col6a2, Col9a1, Col9a3, Creb1, Creb3l2, Creb5, Csf1, Csf1r, Ddit4, Efna1, Erbb3, Erbb4, Ereg, Fgf10, Fgf18, Fgf2, Fgf9, Fgfr1, Fgfr2, Flt1, Flt3, Hgf, Igf1r, Igf2, Il6ra, Il7r, Itga4, Itga6, Itga7, Itgav, Kit, Kitl, Lpar3, Magi2, Mapk1, Mcl1, Nras, Ntrk2, Osmr, Pdgfrb, Pik3ca, Pik3cb, Pik3cd, Pik3r3, Ppp2ca, Ppp2r5d, Ppp2r5e, Prkaa1, Prkaa2, Prkca, Prlr, Pten, Raf1, Rbl2, Rela, Reln, Rheb, Rps6kb1, Rptor, Rxra, Sgk1, Sgk3, Sos1, Spp1, Thbs2, Tnc, Tsc1, Vegfa, Vegfb, Ywhab, Ywhag, Ywhah, Ywhaz*

Supplementary Table S3 | Significant enriched GO terms in the miRNAs linked to neurodegeneration.

GO ID	GO Term	Gene Set Size	Counts	Padj
**Biological process**				
GO:0022008	Neurogenesis	1634	428	<0.001
GO:0006366	Transcription by RNA polymerase II	1926	499	<0.001
GO:0009888	Tissue development	1844	458	<0.001
GO:0044093	Positive regulation of molecular function	1576	378	<0.001
GO:0051649	Establishment of localization in cell	1819	410	<0.001
GO:0006468	Protein phosphorylation	1766	395	<0.001
GO:0012501	Programmed cell death	1874	419	<0.001
GO:0007049	Cell cycle	1544	326	<0.001
GO:0006811	Ion transport	1558	315	<0.001
GO:0042592	Homeostatic process	1809	355	<0.001
**Molecular function**				
GO:0019787	Ubiquitin-like protein transferase activity	403	110	<0.001
GO:0019904	Protein domain specific binding	752	196	<0.001
GO:0008092	Cytoskeletal protein binding	897	215	<0.001
GO:0008270	Zinc ion binding	673	151	<0.001
GO:0003677	DNA binding	1980	423	<0.001
GO:0032553	Ribonucleotide binding	1804	346	<0.001
GO:0005215	Transporter activity	1118	214	<0.001
GO:0005102	Signaling receptor binding	1547	291	<0.001
GO:0042802	Identical protein binding	1803	332	<0.001
GO:0098772	Molecular function regulator	1661	296	<0.001
**Cellular component**				
GO:0030054	Cell junction	1080	267	<0.001
GO:0097458	Neuron part	1906	462	<0.001
GO:0044451	Nucleoplasm part	1021	217	<0.001
GO:0031982	Vesicle	1858	394	<0.001
GO:0031984	Organelle subcompartment	1454	304	<0.001
GO:1902494	Catalytic complex	1280	256	<0.001
GO:0005783	Endoplasmic reticulum	1644	321	<0.001
GO:0005730	Nucleolus	885	162	<0.001
GO:0031226	Intrinsic component of plasma membrane	1492	246	<0.001
GO:0044430	Cytoskeletal part	1570	258	<0.001
